# Supplementary figures and images for: Symbiotic Versatility in Action: Trebouxia Diversity Expands the Niche of the Lichen Xanthoria parietina
Source: Environ Microbiol. 2026 Jul 16;28(7):e70379. doi: 10.1111/1462-2920.70379 (PMC13375602; doi:10.1111/1462-2920.70379)

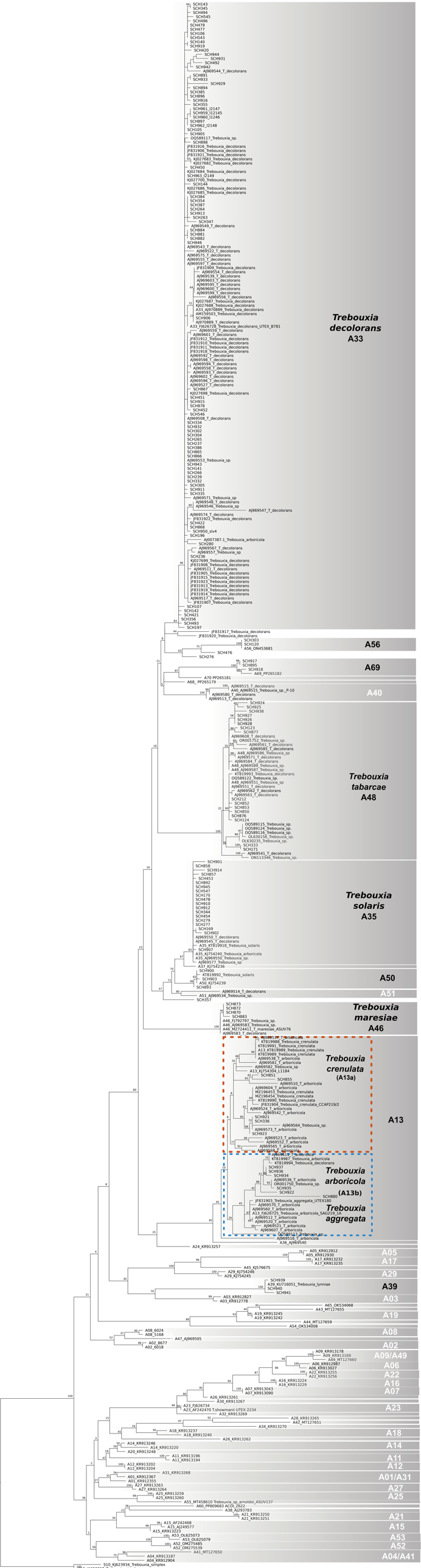

Supplement: Supplementary file 1 — Figure S1: RAxML vertical phylogeny based on nrITS sequence data, depicting the phylogenetic relationships among Trebouxia phycobionts of the analysed Xanthoria species. [file EMI-28-e70379-s011.pdf]

Symbiotic partner accumulation curves

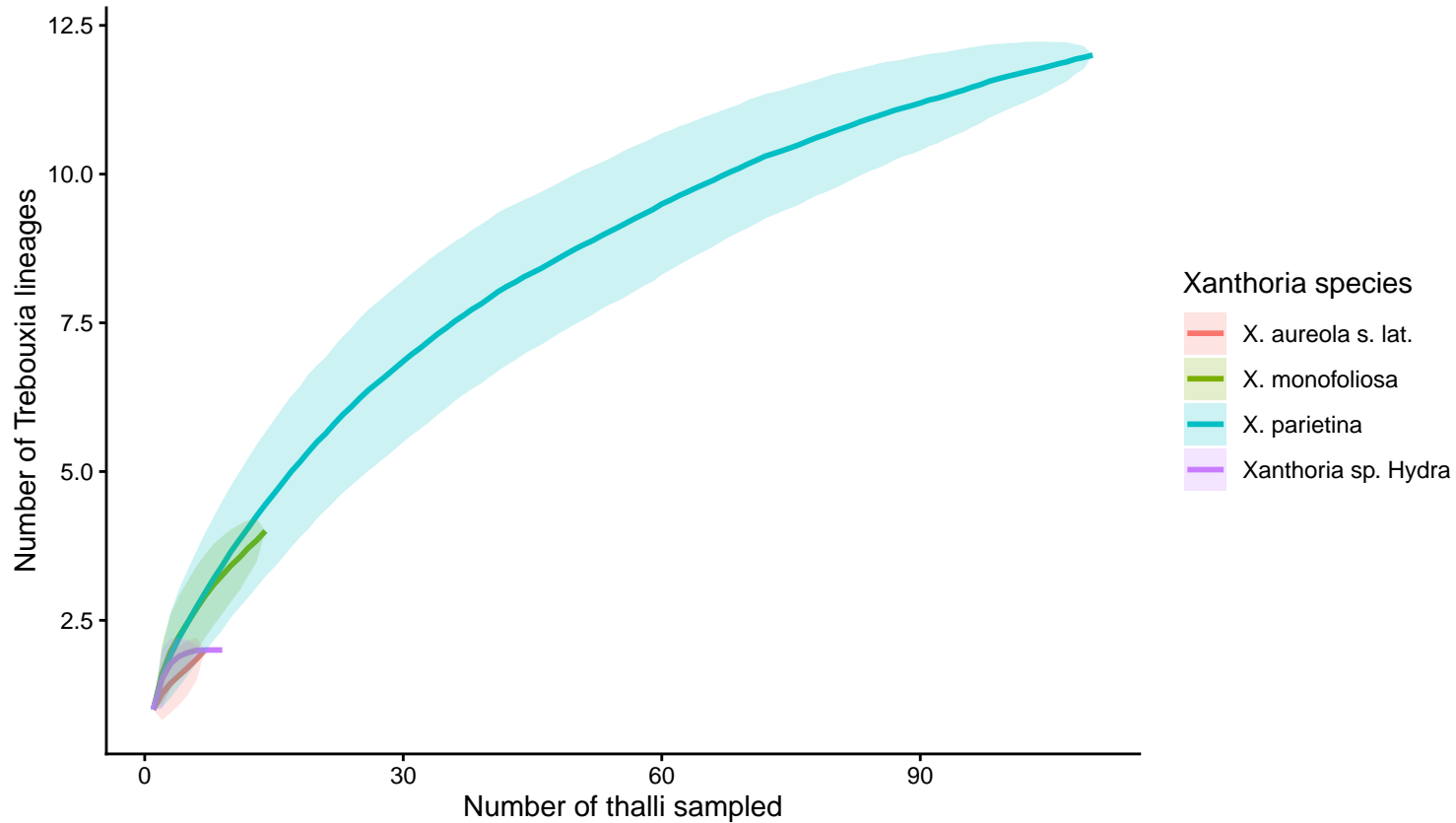

Supplement: Supplementary file 2 — Figure S2: Rarefaction curves showing phycobiont richness as a function of sampling effort for Xanthoria species. [file EMI-28-e70379-s009.pdf]

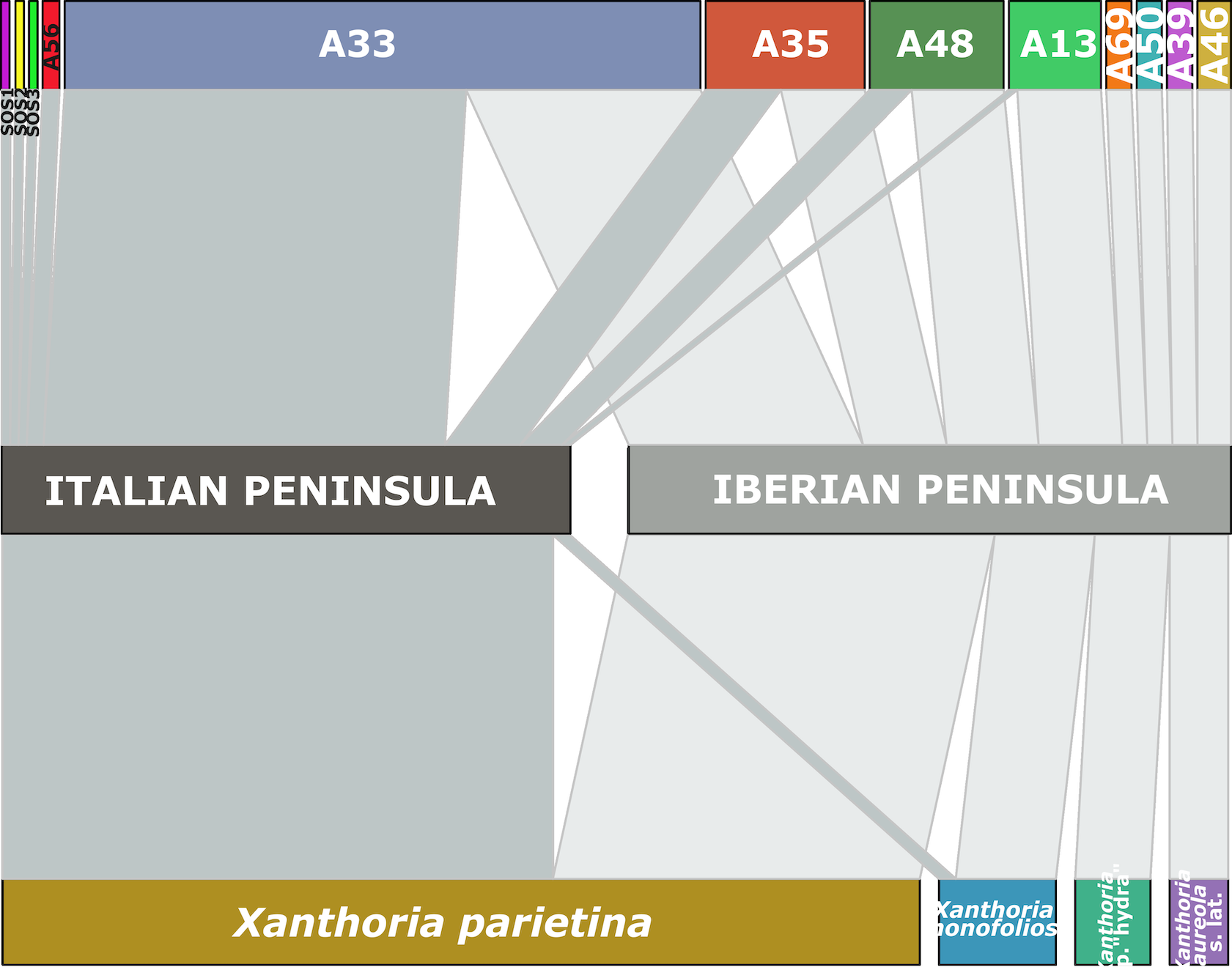

Supplement: Supplementary file 3 — Figure S3: Tripartite network combining the bipartite networks mycobiont versus region (ItP or IbP) and phycobiont versus region (ItP or IbP). The top boxes represent the phycobionts detected in Xanthoria spp. The geographical regions are shown in the middle boxes. The basal boxes show the Xanthoria mycobiont species. The width of the links is proportional to the number of specimens representing the associations. Top boxes without a Trebouxia lineage code correspond to SOS sequences. [file EMI-28-e70379-s008.tiff]

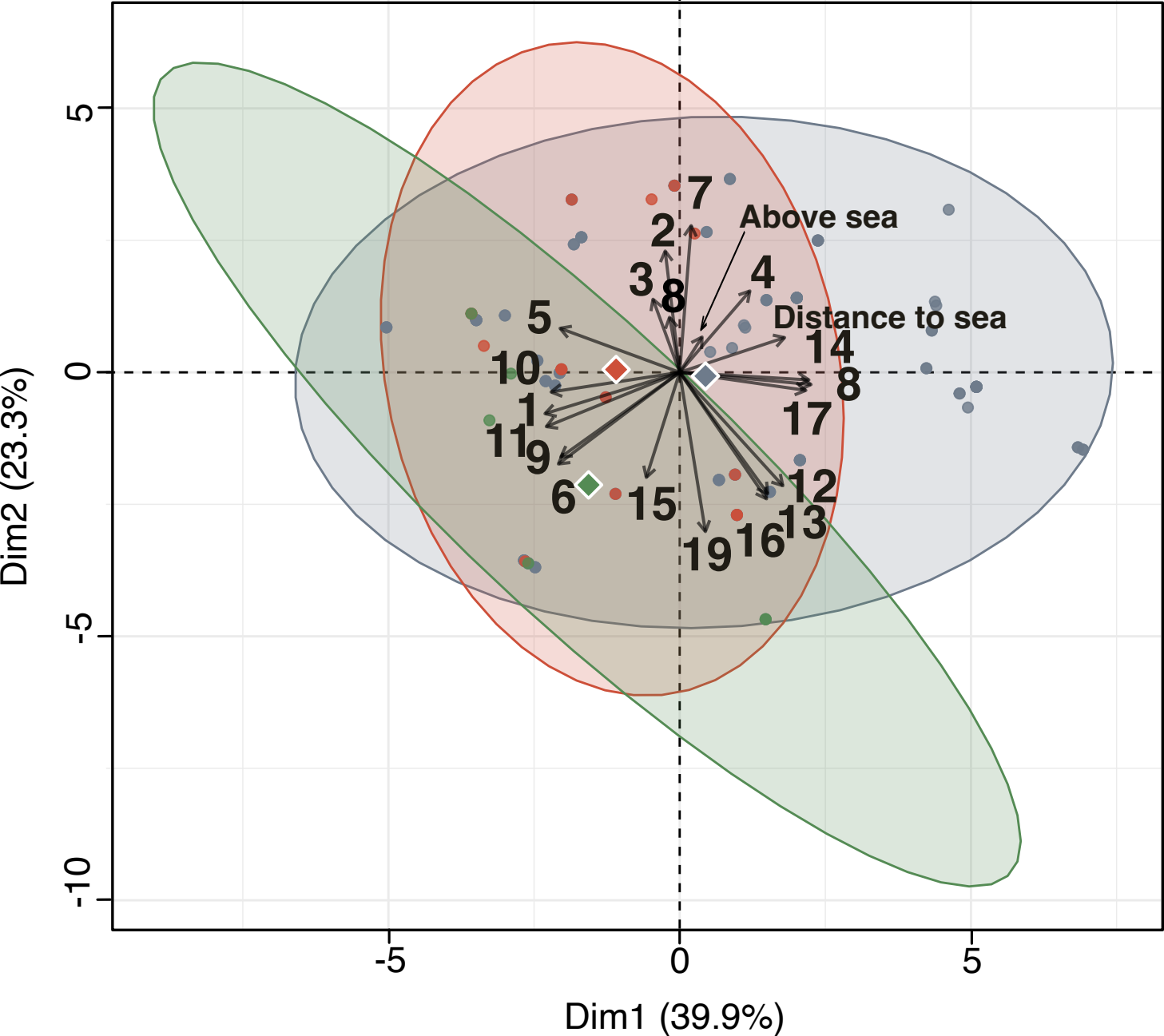

Supplement: Supplementary file 4 — Figure S4: PCA of 19 WorldClim bioclimatic variables, along with altitude and distance to the sea of Trebouxia decolorans (A33), T. solaris (A35) and T. tabarcae (A48). [file EMI-28-e70379-s010.pdf]

***Trebouxia decolorans***

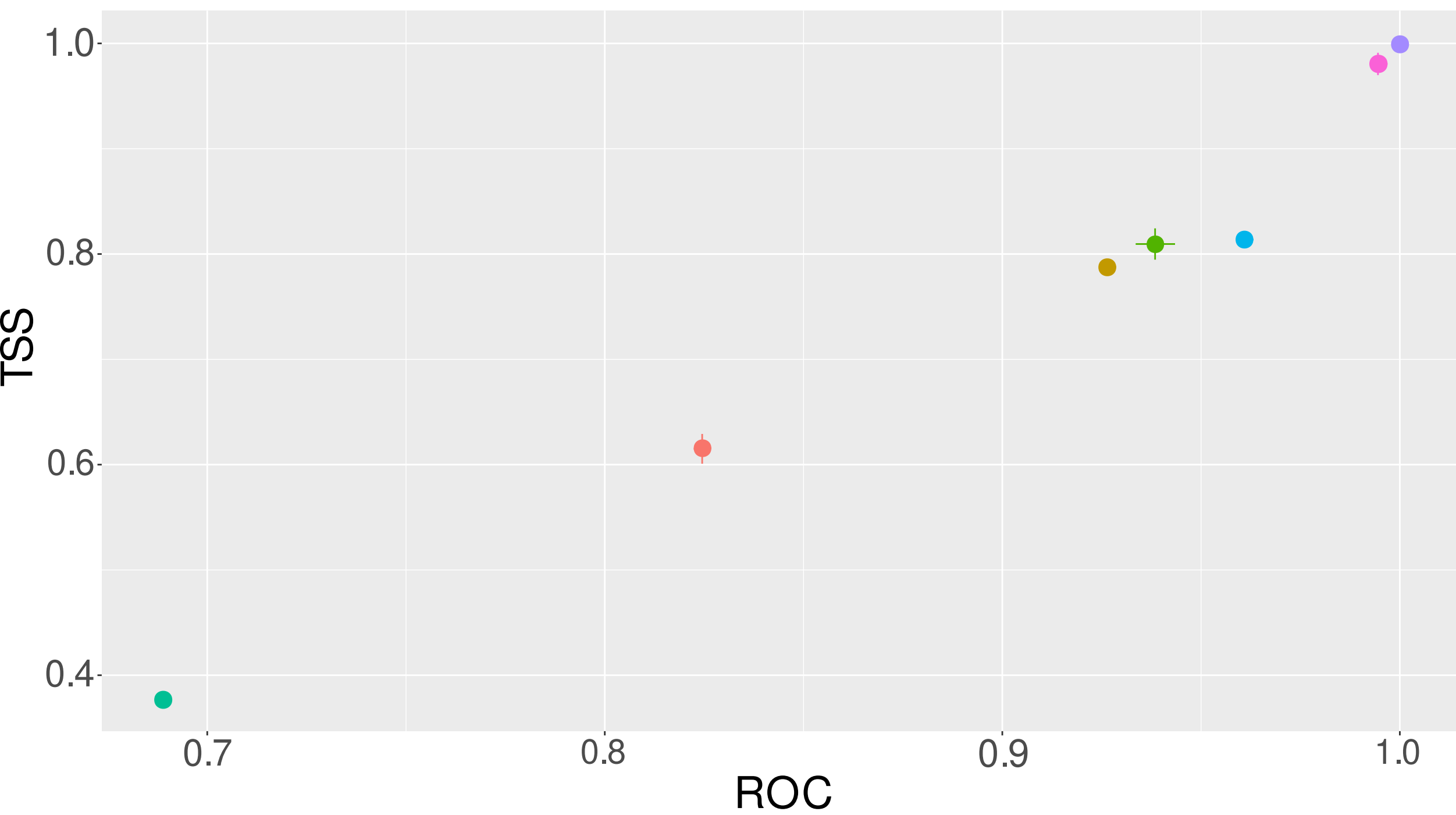

***Trebouxia solaris***

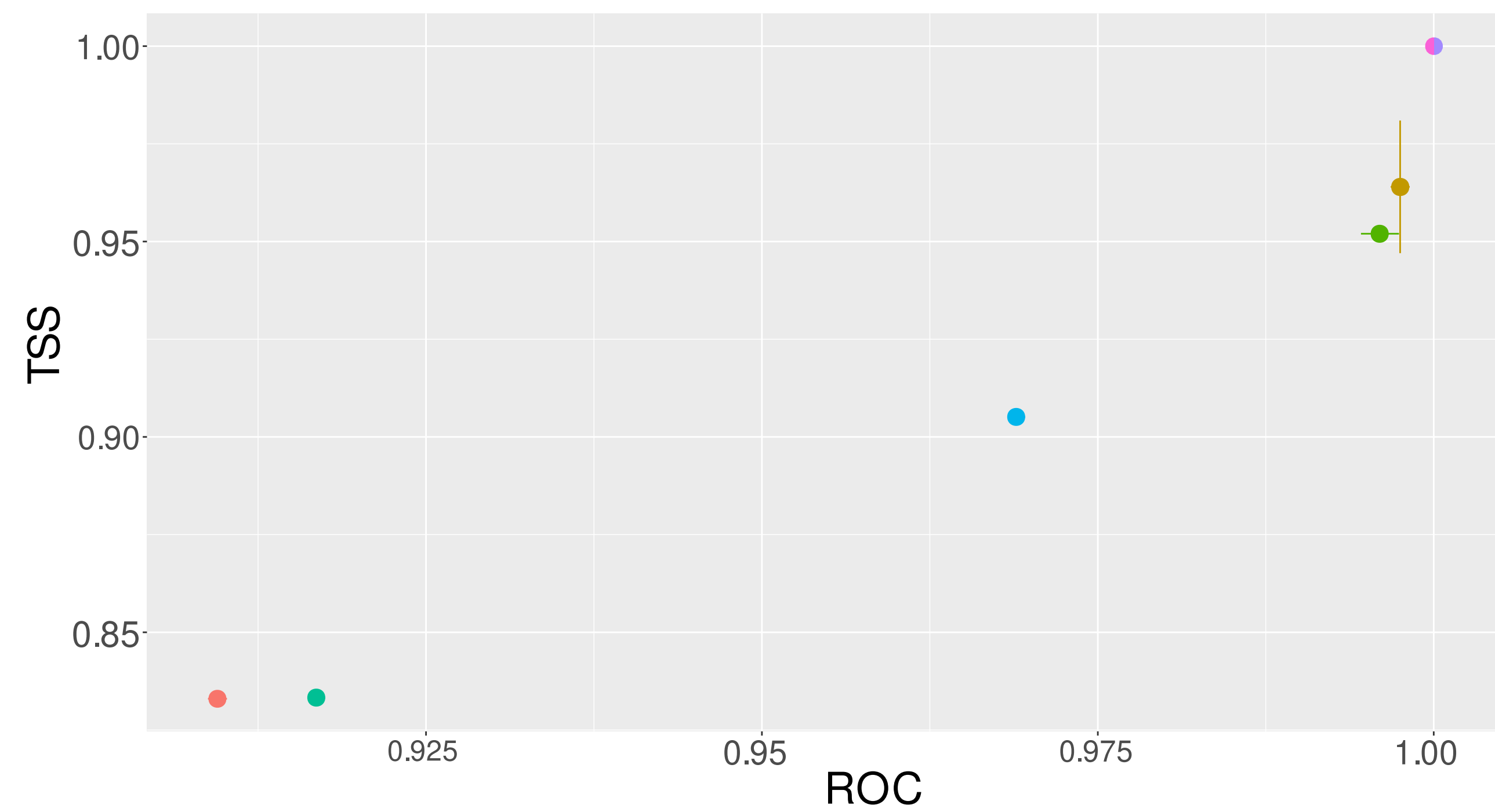

***Trebouxia tabarcae***

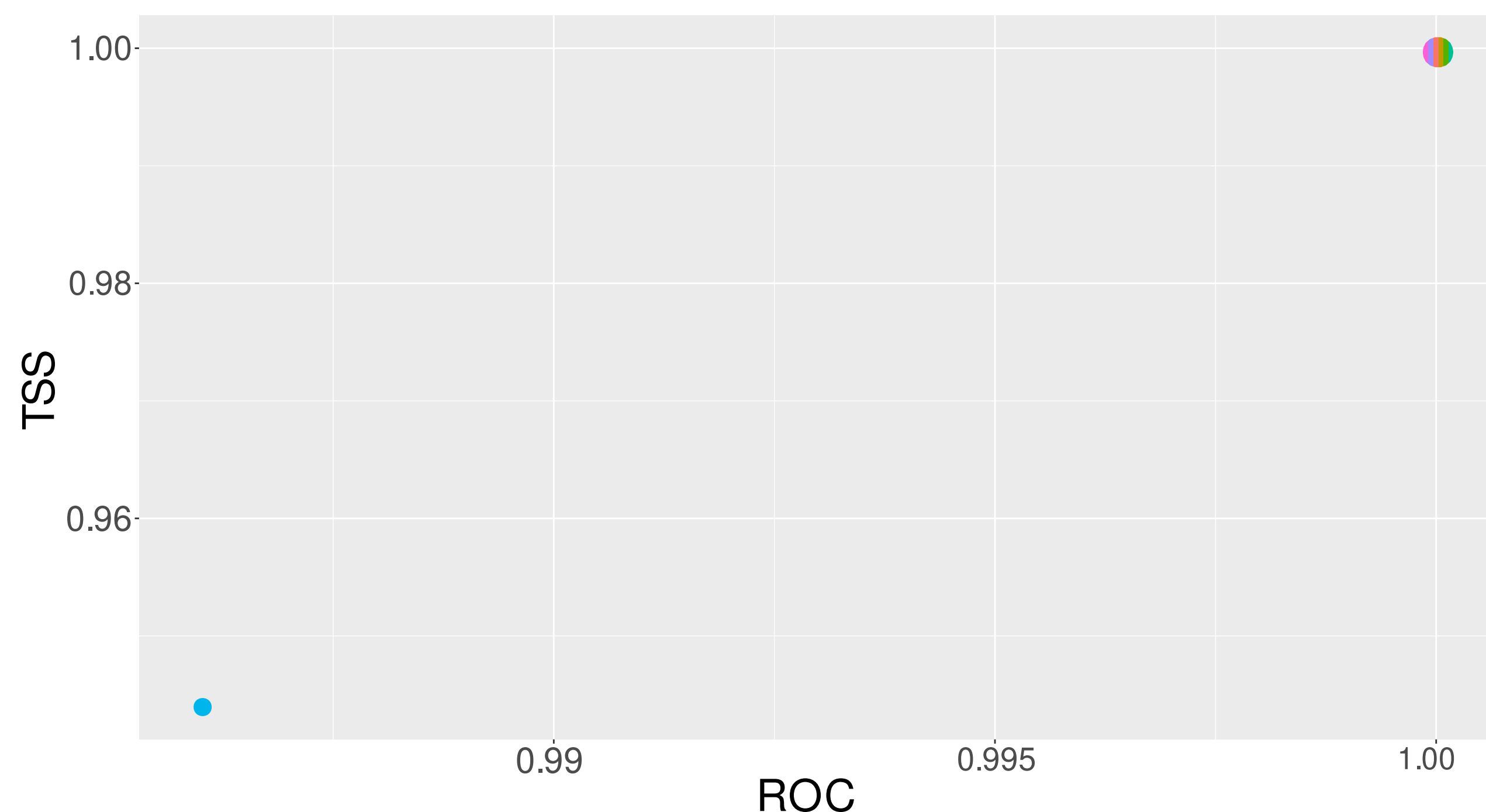

**Models:**

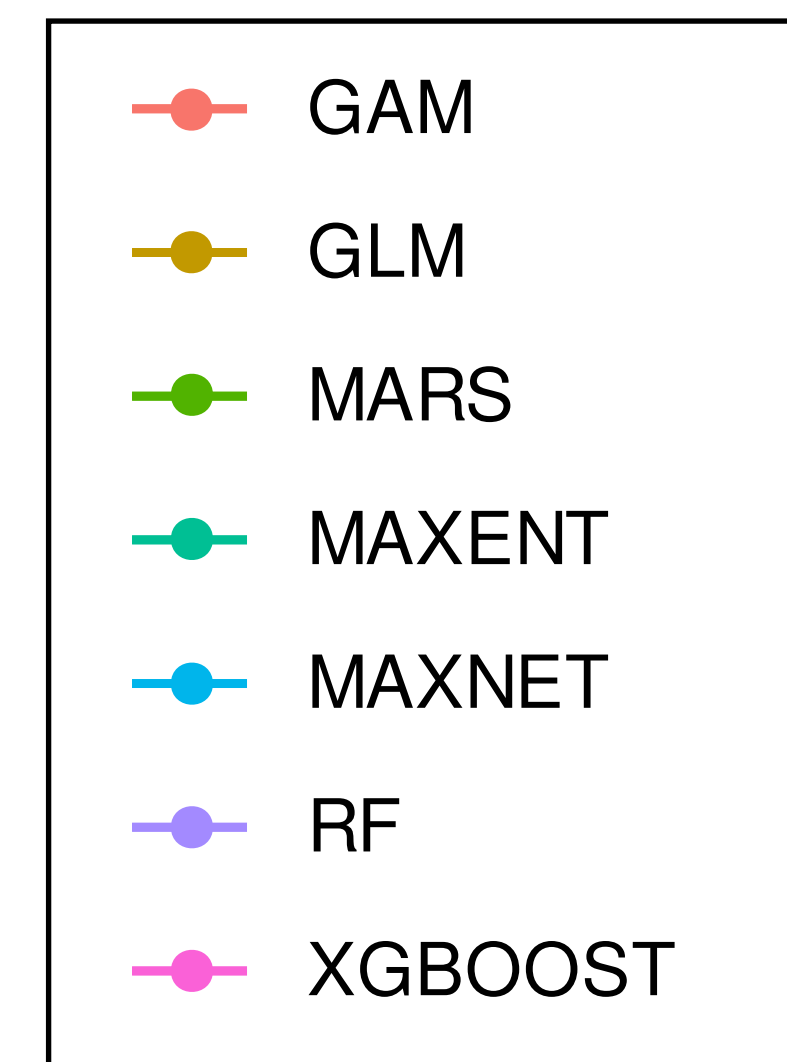

Supplement: Supplementary file 6 — Figure S6: Evaluation of model performance for seven distribution modelling methods (MAXENT, MAXNET, GAMs, GLMs, MARS, RF and XGBOOST) applied to Trebouxia decolorans (A33), T. solaris (A35) and T. tabarcae (A48). Model performance was assessed using Receiver Operating Characteristic (ROC) curves and True Skill Statistic (TSS) scores. ROC/TSS values above 0.80 indicate consistently high predictive accuracy across all models. [file EMI-28-e70379-s001.pdf]

*Trebouxia decolorans* (A33)

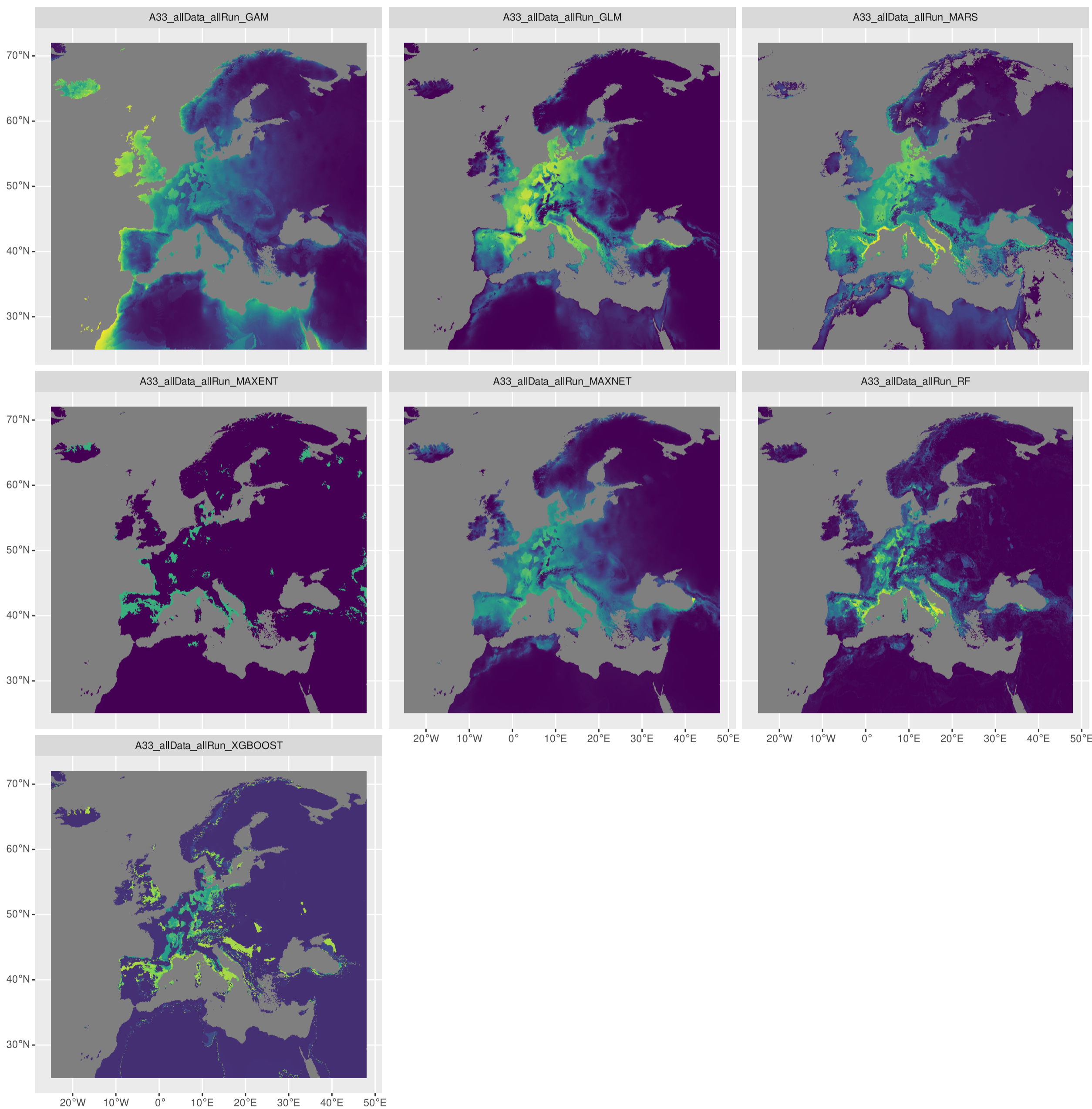

*Trebouxia solaris* (A35)

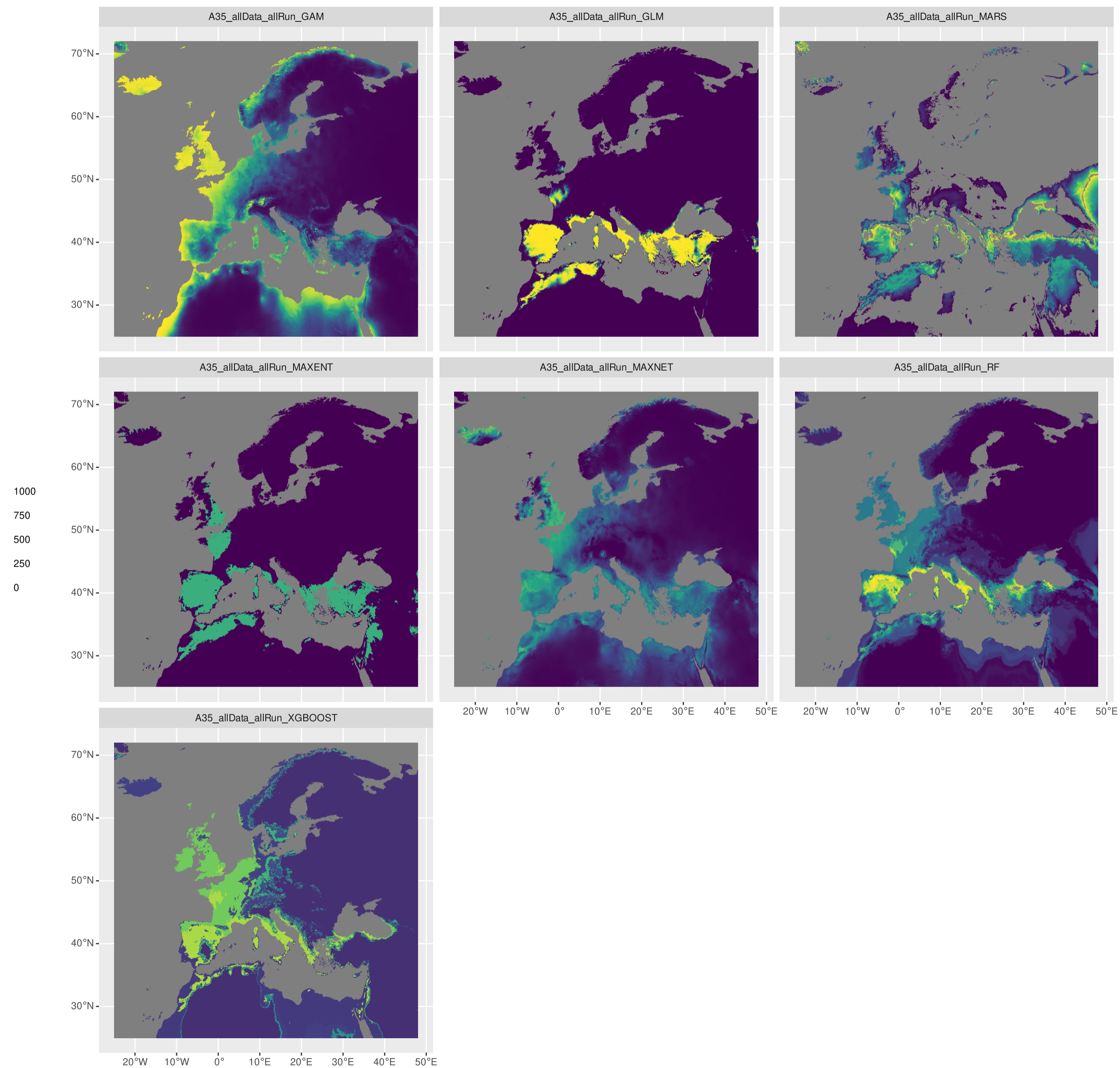

*Trebouxia tabarcae*

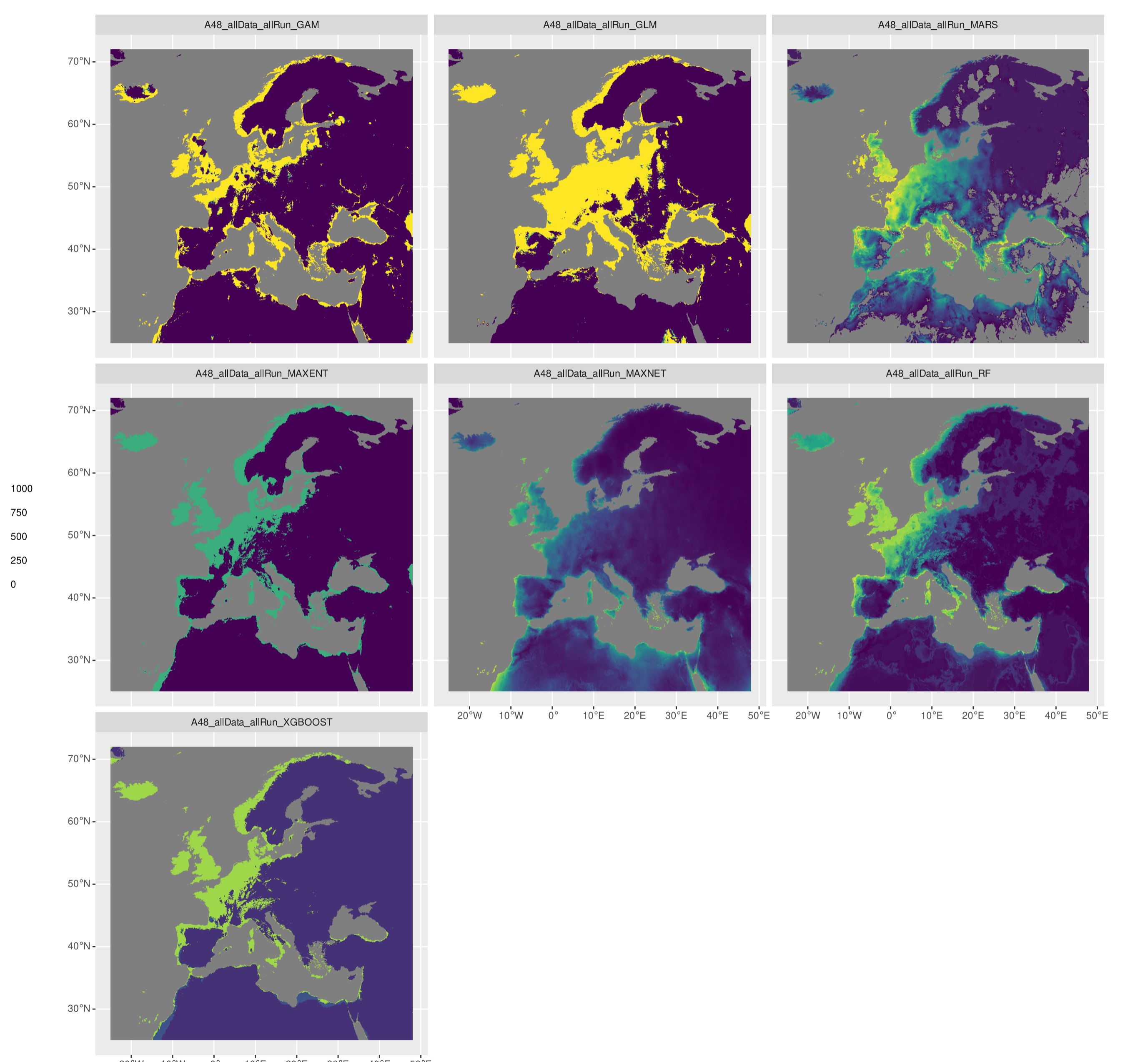

Supplement: Supplementary file 7 — Figure S7: Potential distribution of Trebouxia decolorans (A33), T. solaris (A35) and T. tabarcae (A48) estimated by different modelling methods (MAXENT, MAXNET, GAMs, GLMs, MARS, RF and XGBOOST), using manual selection of bioclimatic variables. [file EMI-28-e70379-s004.pdf]

## *Trebouxia decolorans*

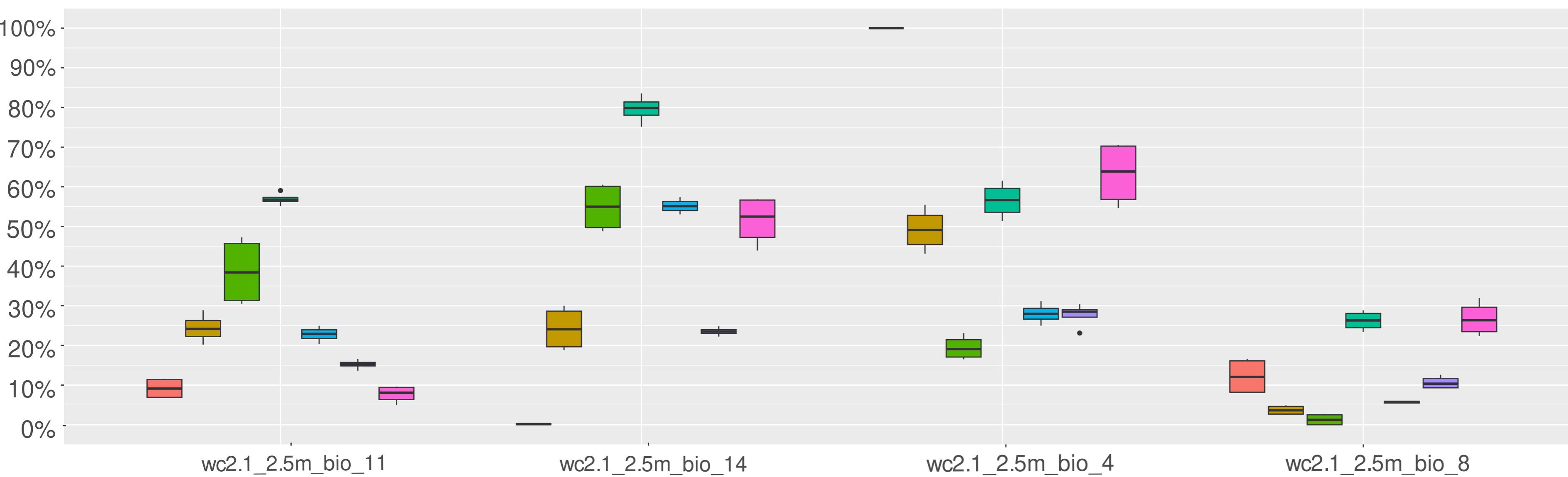

## *Trebouxia solaris*

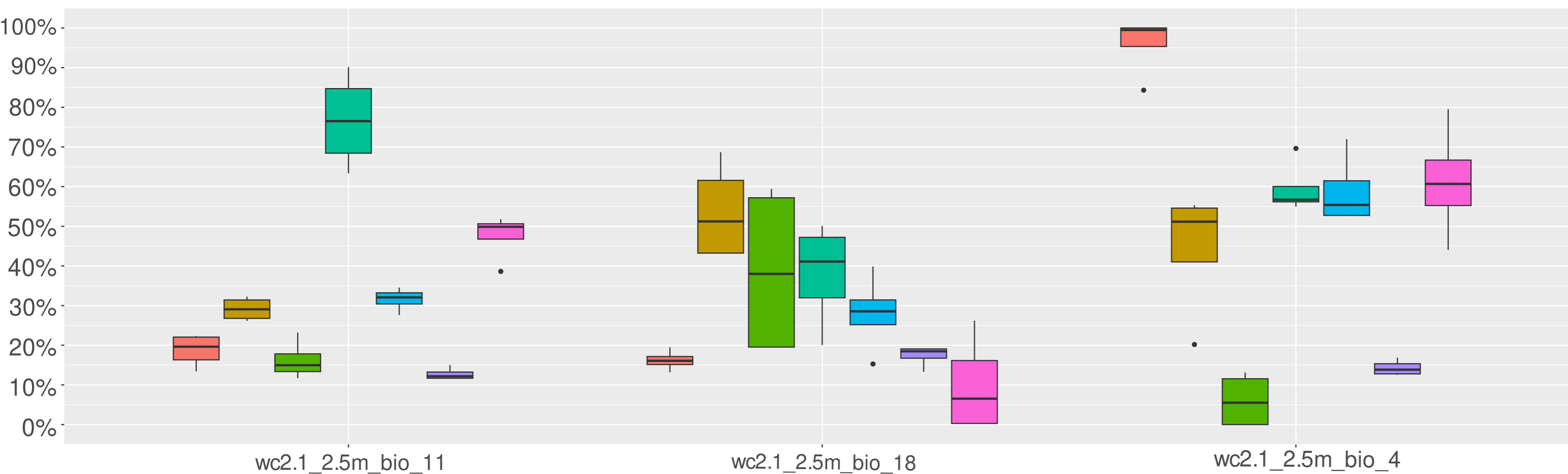

### Models:

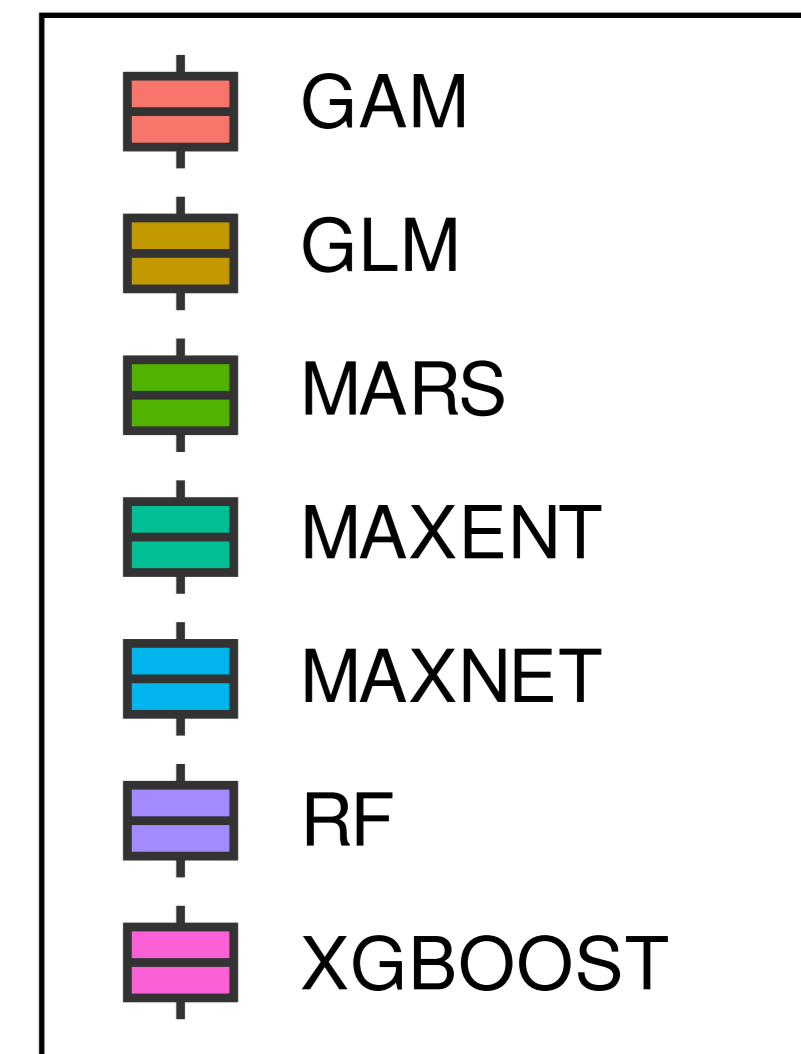

## *Trebouxia tabarcae*

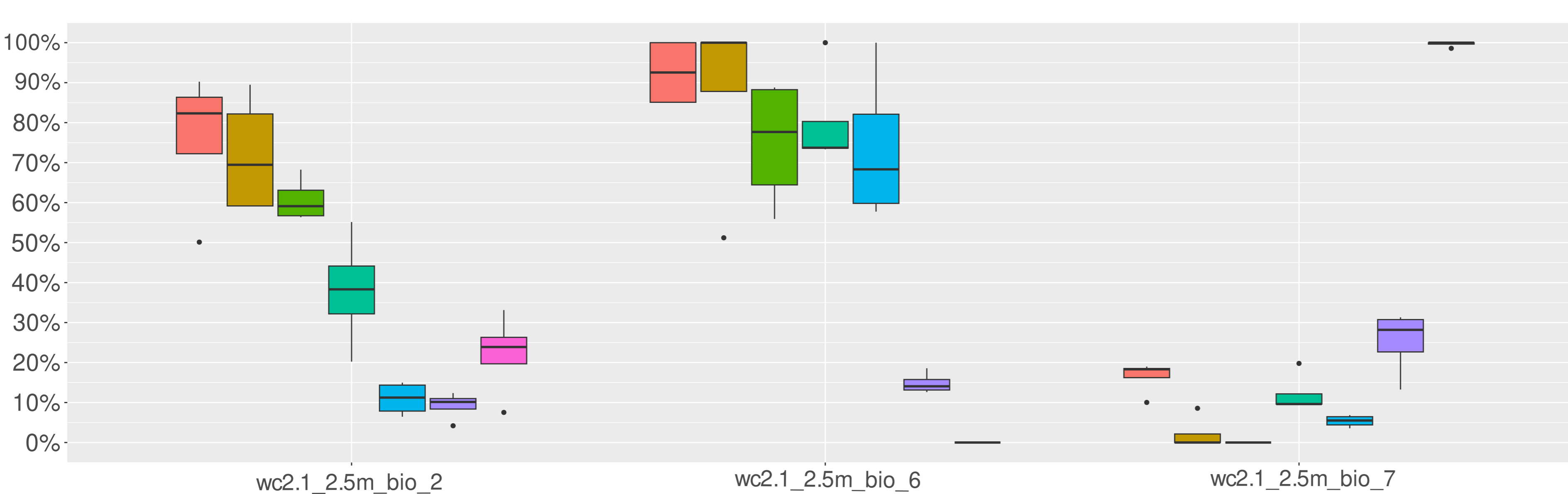

Supplement: Supplementary file 8 — Figure S8: Relative contribution of each selected bioclimatic variable to the seven distribution modelling algorithms applied to Trebouxia decolorans (A33), T. solaris (A35) and T. tabarcae (A48). [file EMI-28-e70379-s003.pdf]
