# Supplementary material for: Symbiotic Versatility in Action: Trebouxia Diversity Expands the Niche of the Lichen Xanthoria parietina
Source: Environ Microbiol. 2026 Jul 16;28(7):e70379. doi: 10.1111/1462-2920.70379 (PMC13375602; doi:10.1111/1462-2920.70379)
Supplement: Supplementary file 5 — Figure S5: Boxplots showing the range of values of the selected bioclimatic variables for the distribution modelling analysis of Trebouxia decolorans (A33), T. solaris (A35) and T. tabarcae (A48). [file EMI-28-e70379-s006.pdf]

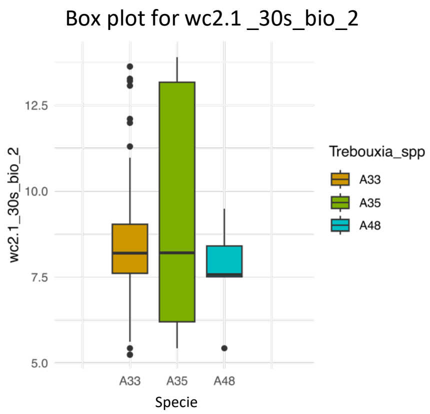
Box plot for wc2.1 _30s_bio_2
wc2.1_30s_bio_2
12.5
10.0
7.5
5.0
A33
A35
A48
Specie
Trebouxia_spp
A33
A35
A48

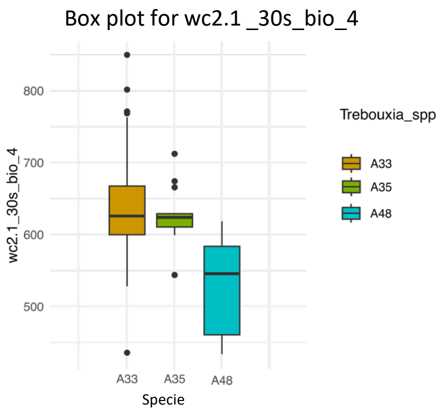
Box plot for wc2.1 _30s_bio_4
wc2.1_30s_bio_4
800
700
600
500
A33
A35
A48
Specie
Trebouxia_spp
A33
A35
A48

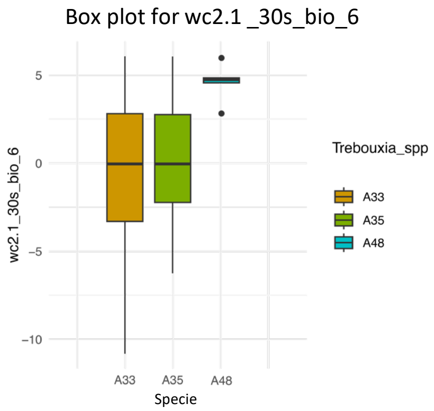
Box plot for wc2.1 _30s_bio_6
wc2.1_30s_bio_6
5
0
−5
−10
A33
A35
A48
Specie
Trebouxia_spp
A33
A35
A48

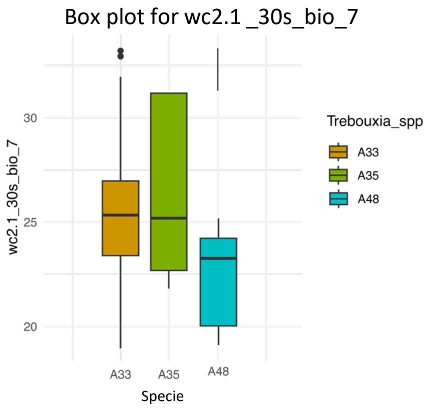
Box plot for wc2.1 _30s_bio_7
wc2.1_30s_bio_7
30
25
20
A33
A35
A48
Specie
Trebouxia_spp
A33
A35
A48

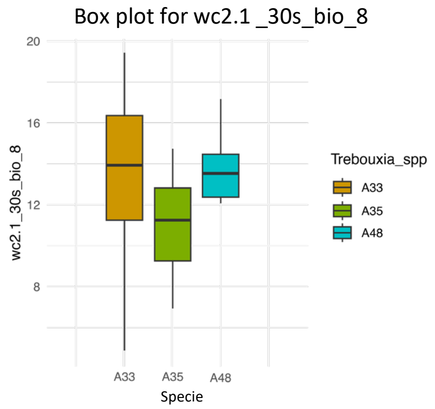
Box plot for wc2.1 _30s_bio_8
wc2.1_30s_bio_8
20
16
12
8
A33
A35
A48
Specie
Trebouxia_spp
A33
A35
A48

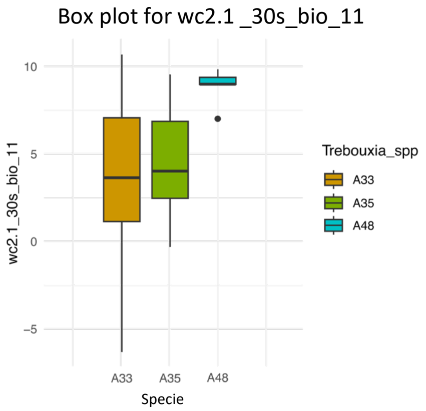
Box plot for wc2.1 _30s_bio_11
wc2.1_30s_bio_11
10
5
0
−5
A33
A35
A48
Specie
Trebouxia_spp
A33
A35
A48

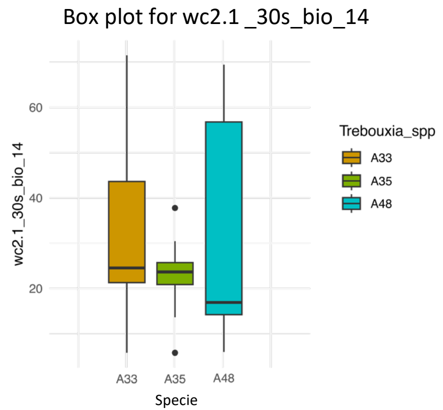
Box plot for wc2.1 _30s_bio_14
wc2.1_30s_bio_14
60
40
20
A33
A35
A48
Specie
Trebouxia_spp
A33
A35
A48

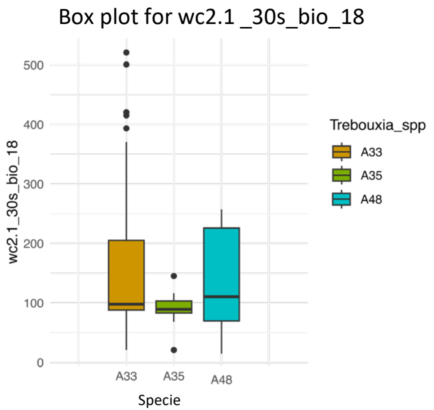
Box plot for wc2.1 _30s_bio_18
wc2.1_30s_bio_18
500
400
300
200
100
0
A33
A35
A48
Specie
Trebouxia_spp
A33
A35
A48
